# Risk Factors for *Cryptococcus gattii* Infection, British Columbia, Canada

**DOI:** 10.3201/eid1702.101020

**Published:** 2011-02

**Authors:** Laura MacDougall, Murray Fyfe, Marc Romney, Mike Starr, Eleni Galanis

**Affiliations:** Author affiliations: British Columbia Centre for Disease Control, Vancouver, British Columbia, Canada (L. MacDougall, M. Fyfe, M. Romney, M. Starr, E. Galanis);; Public Health Agency of Canada, Ottawa, Ontario (L. MacDougall);; Vancouver Island Health Authority, Victoria, British Columbia, Canada (M. Fyfe);; University of British Columbia, Vancouver (M. Fyfe, M. Romney, E. Galanis);; St. Paul’s Hospital, Vancouver (M. Romney);; Royal Children’s Hospital, Melbourne, Victoria, Australia (M. Starr)

**Keywords:** Cryptococcus neoformans, Cryptococcus gattii, cryptococcosis, respiratory infection, fungi, parasites, case–control study, corticosteroids, risk factor, research

## Abstract

To determine whether particular environmental, medical, or behavioral risk factors existed among *Cryptcoccus gattii*–infected persons compared with the general population, we conducted a sex-matched case−control study on a subset of case-patients in British Columbia (1999–2001). Exposures and underlying medical conditions among all case-patients (1999–2007) were also compared with results of provincial population–based surveys and studies. In case−control analyses, oral steroids (matched odds ratio [MOR] 8.11, 95% confidence interval [CI] 1.74–37.80), pneumonia (MOR 2.71, 95% CI 1.05–6.98), and other lung conditions (MOR 3.21, 95% CI 1.08–9.52) were associated with infection. In population comparisons, case-patients were more likely to be >50 years of age (p<0.001), current smokers (p<0.001), infected with HIV (p<0.001), or have a history of invasive cancer (p<0.001). Although *C. gattii* is commonly believed to infect persons with apparently healthy immune systems, several immunosuppressive and pulmonary conditions seem to be risk factors.

*Cryptococcus gattii* emerged on Vancouver Island, British Columbia (BC), Canada, in 1999, resulting in one of the highest incidences of this infection worldwide (*1**,**2*). The natural reservoir of *C. gattii* seems to be soil and plant debris, and it has been associated with numerous tree species (*3**,**4*). When inhaled, this encapsulated basidiomycetous yeast may infect humans as well as diverse animal species (*5*). Infected humans may be asymptomatic but usually exhibit pulmonary infection characterized by cough, shortness of breath, and single or multiple pulmonary nodules visible on radiographs of the lung. Body sites such as brain, skin, and bone are affected less commonly (*6**,**7*). Approximately 18% of patients in British Columbia have disseminated disease, including meningitis and brain cryptococcomas (*1*). The case-fatality rate among BC residents during 1999–2007 was 8.7% (*1*).

*C. gattii* is believed to infect persons with uncompromised immune systems (*8**,**9*), unlike *C. neoformans,* a relatively common opportunistic pathogen in HIV-infected patients and other immunocompromised patients. Although true differences in species-specific pathogenicity may exist, *C. gattii* may also commonly infect persons with undetected immune deficiencies. Early studies have identified several risk factors for *Cryptococcus* spp. infection; however, these do not distinguish between species. In experimental studies, steroids have been shown to decrease host resistance to infection (*10*). Case-series investigations have linked corticosteroid use with an increased risk of dying from cryptococcal meningitis (*11*) and with an increased risk for disseminated disease (*12*). A nationwide survey of 163 HIV-negative cryptococcosis patients in France (1985–1993) showed that having malignancies (32%), undergoing organ transplantation (19%), and receiving corticosteroid therapy (33%) were the main predisposing factors (*13*). Other frequently identified predisposing conditions for cryptococcosis include sarcoidosis, hyper-immunoglobulin (Ig) M and hyper–IgE syndromes, and CD4+ T-cell lymphopenia in those who are HIV negative (*14*). Case reports have also suggested that cirrhosis is a risk factor for cryptococcal peritonitis (*15*).

Compared with results for adults, cryptococcal infections are rare in children, no matter their HIV status (*16*). Historical studies of cryptococcosis patients have shown that higher proportions of male patients had the disease, both before and after the HIV epidemic (*14*). The risk for disseminated infection among HIV-positive persons with cryptococcal infection was almost 4× higher for those who smoked at the time of diagnosis than for those who did not (*17*).

Many studies that examined predisposing factors for *Cryptococcus* infection in humans have done so in selected populations (e.g., HIV-positive patients), and assessment has been frequently limited to medical rather than behavioral or environmental exposures. Often laboratory testing was not undertaken to distinguish patients with *C. gattii* infection from those with *C. neoformans* infection. In newer studies, which have obtained subtyping information, typically, the number of *C. gattii* isolates was insufficient to determine risk factors. Risk factors suggested in the medical literature have arisen from case reports and case series that described the proportion of patients with particular underlying conditions. Although some investigations have compared risk factors between patients with *C. gattii* and those with *C. neoformans* infections (*8**,**9*), we could find no examples in which case-patients were compared with healthy controls to determine risks for disease acquisition.

Ecologically, epidemiologically, and clinically, *C. gattii* is sufficiently different from its fungal relative *C. neoformans* (including *C. neoformans* var. *grubii* and *C*. *neoformans* var. *neoformans*) to warrant its own species designation (*18*). It therefore seems reasonable to assume that the risk factors for disease acquisition may not be the same. We undertook this current investigation to determine whether, compared with the general population, particular medical, behavioral, or environmental risk factors existed among case-patients with *C. gattii* infection in British Columbia, Canada.

## Methods

Risk factors for *C. gattii* infection were evaluated in 2 ways. First, a case–control study was conducted on a subset of case-patients (1999–2001) to examine which medical and environmental exposures increased the odds of infection. Second, risk behaviors and underlying medical conditions of all case-patients (1999–2007) were compared with those of the general BC population by using existing data from population-based surveys and studies.

### Case Ascertainment

Patients who became study participants included those with culture-confirmed *C. gattii* infection (for which genotyping had been done) as well as those with laboratory evidence of cryptococcal infection (determined from antigen detection and histopathologic or microscopic examination) who were also HIV negative and had been exposed to a local *C. gattii*–endemic area in the year before disease onset (*1**,**2**)*. HIV status and geographic exposure were included as part of the case definition for patients from whom culture results were unavailable because hospitalization rates had increased sharply in this population group in 1999, signaling the onset of the *C. gattii* outbreak in British Columbia (*2*). Information on case-patients was obtained from the BC Cryptococcus Database, which contains laboratory and interview data from patients with a diagnosis since 2001 when infection with *Cryptococcus* species became reportable. All available isolates are routinely sent to the British Columbia Centre for Disease Control Public Health Microbiology and Reference Laboratory for culturing, serotyping, and molecular characterization. Cases diagnosed before 2001 were identified either through reporting by laboratory physicians or through a review of the Provincial Electronic Hospital Separation Database for the years 1995–2001 for records containing the International Classification of Diseases, 9th Revision (ICD-9), code 117.5 (cryptococcosis) without ICD-9 codes V08 and 042.X (HIV/AIDS). Stored clinical isolates were also typed retrospectively (*2*). Specimens were identified as *C. gattii* as previously described (*19**–**21*).

### Case−Control Study

Thirty-eight BC residents whose *C. gattii* infections were diagnosed from January 1999 through December 2001 were eligible for inclusion in the case–control study. Two controls per case-patient were identified through the general practitioners of the infected persons. Controls were matched with case-patients by sex since this was considered a likely confounder for many behavioral risk factors. Controls were required to have had a chest radiograph showing absence of active pulmonary disease after their matched case-patient’s date of diagnosis.

Case-patients and controls were interviewed twice by using structured, in-depth questionnaires. The first interview was conducted face to face at the participants’ homes. Information collected included demographic variables; medical history (e.g., has a doctor ever diagnosed you with X?), smoking status; clinical symptoms; occupation; travel history; recreational activities; gardening and landscaping activities; construction activities; and exposure to botanical gardens, zoos, aquariums, agriculture, animals, compost, bark mulch, various tree species, and wooded areas. Case-patients were asked to consider activities in the 3 months before onset of symptoms. Controls were interviewed about the exposure period corresponding to their matched case-patient. Current smokers were those who self-identified as smokers of cigarettes or cigars at the time of interview. Respondents who had ever smoked were persons who had smoked cigarettes or cigars on a regular basis at some point in their lives.

Frequency analysis was performed by using SPSS version 10.0 (SPSS Inc, Chicago, IL, USA). Matched odds ratios and 95% confidence intervals were calculated by using S-Plus version 6.0 (MathSoft Inc, Seattle, WA, USA). Stratified analyses were conducted to explore potential confounding relationships. Logistic regression was not possible because of the collinearity of the data and small sample size.

### Population Comparison

Information on a smaller number of risk factors (than obtained from the questionnaire) was also routinely collected from case-patients, their physicians, or both, during standardized public health interviews of all BC case-patients from 1999 through 2007. Data were collected from symptomatic and asymptomatic case-patients. Past medical conditions were included; steroid use was evaluated over the 3-month period before onset of infection for case-patients whose conditions were diagnosed during 1999–2001 and over a 12-month period for later identified case-patients. The proportion of *C. gattii*–infected case-patients was calculated for the following age groups: 0–19 20–39, 40–49, 50–59, 60–69, 70–79, and >80 years. Proportions were also tabulated of case-patients who were male, were current smokers at the time of interview, were infected with HIV, had chronic obstructive pulmonary disease (COPD) or asthma, had taken systemic corticosteroids, or had a history of invasive cancer. Invasive cancer included all cancers reported, including melanoma (but no other skin cancers). Prevalence proportions were calculated among case-patients for whom data on a particular risk factor were available. Prevalence proportions for *C. gattii* case-patients were then compared with prevalence proportions for the general BC population obtained from existing data sources. These included provincial statistics on age and sex distribution (*22*), BC smoking prevalence estimates from a national community health survey (*23*), and type-specific cancer prevalence from the BC Cancer Agency (*24*). Provincial estimates of HIV prevalence, derived by previously published methods (*25*), were provided by BC Centre for Disease Control (M. Gilbert, pers. comm.). We used the χ^2^ test for small dependent populations in Microsoft Excel (Microsoft Corp., Redmond, WA, USA) to compare prevalence of risk factors among *C. gattii* case-patients with the overall BC population.

## Results

### Case–Control Study

During 1999–2001, 38 case-patients met the case definition criteria for inclusion in the case–control study. Nineteen cases were diagnosed by culture, the remaining 19 by histopathologic examination. Isolates from 18 case-patients with culture-confirmed infection were *C. gattii*, serotype B; 1 isolate could not be retrieved for subtyping. None of the 38 patients was HIV positive. The mean age at diagnosis was 59.7 years (range 20–82 years; SD 13.49); 22 were male. Thirty-six case-patients were Caucasian, and 2 were of Asian descent, which is consistent with provincial ethnicity statistics (data not shown). Thirty were retired or unemployed at the time of their illness. Ten case-patients had cryptococcal meningitis when they sought treatment; the remainder exhibited respiratory infection. The most common self-reported symptoms were cough (21 patients), shortness of breath (20 patients), night sweats (20 patients), and fever (20 patients).

Thirty matched sets of case-patients and controls were interviewed (8 case-patients could not be matched). No significant difference in age was found (p = 0.24). Case-patients were more likely than controls to report having ever received a physician’s diagnosis of pneumonia (matched odds ratio [MOR] 2.71, 95% confidence interval [CI] 1.05–6.98) or other lung conditions (MOR 3.21, 95% CI 1.08–9.52) (Table 1). Case-patients were also more likely than controls to have taken systemic corticosteroids (MOR 8.11, 95% CI 1.74–37.80), including prednisone (12 case-patients) and methylprednisolone (1 case-patient), during the 3 months before their illness. No single indication for steroid therapy predominated among case-patients. Although oral steroid use had the strongest association with *C. gattii* infection, stratified analysis did not identify confounding effects on other variables. Neither smoking nor a history of cancer was associated with illness. Chopping wood, pruning trees, and cleaning up branches were significantly less commonly reported among case-patients than among controls, whereas 1 activity, conducting outdoor repairs to a house or building, was more common among case-patients.

**Table 1:** ORs for risk factors for *Cryptococcus gattii* infection for 30 matched case-patient and general population sets, British Columbia, Canada, 1999–2007*

Risk factors	MOR (95% CI)
Medical	
** Lung conditions†**	**3.21 (1.08–9.52)**
** Pneumonia**	**2.71 (1.05–6.98)**
Asthma	0.45 (0.12–1.66)
Diabetes	0.65 (0.17–2.50)
Anemia	2.64 (0.74–9.44)
Arthritis	0.97 (0.37–2.49)
Liver disease	4.00 (0.36–44.10)
Cancer	2.03 (0.63–6.81)
Other fungal infections	1.69 (0.23–12.20)
Tuberculosis	3.24 (0.29–36.60)
**Oral steroid use**‡	**8.11 (1.74–37.80)**
Current smoker	1.00 (0.34–2.93)
Ever smoked	1.18 (0.44–3.20)
Environmental‡§	
Living with 1 mile of woods	1.70 (0.17–2.02)
Outdoor building or repairing house	4.00 (1.00–16.00)
** Cutting/chopping wood**	**0.17 (0.04–0.76)**
** Pruning**	**0.28 (0.09–0.88)**
** Cleaning up branches**	**0.29 (0.10–0.84)**
Digging earth	0.93 (0.38–2.30)
Camping	1.23 (0.23–2.91)
Gardening	1.15 (0.47–2.79)

*MOR, matched odds ratio; CI, confidence interval. **Boldface** indicates significant risk factors. †Includes emphysema, chronic bronchitis, chronic obstructive pulmonary disease, sarcoidosis. ‡In 3 mo before symptom onset. §Other environmental risk factors not significant at α = 0.05: animal or crop farm within 1 mile of residence; construction or landscaping activities; cleaning of buildings, eaves, troughs, or bird feeders; contact with individual tree species; visits to botanical gardens; boating; use of compost materials and bark mulch.

### Population Comparison

Among 218 *C. gattii* case-patients reported in British Columbia from 1999–2007, 124 (56.9%) had a culture-confirmed infection. The mean age was 58.7 years. Of case-patients with risk factor information available, 65 (41.9%) of those >12 years of age smoked at the time of diagnosis, 6 (3.7%) had HIV infection, and 38 (24.7%) had a history of invasive cancer. The most common forms of cancer reported were leukemia (n = 7), lymphoma (n = 6), and lung (n = 6). Seventy (38.0%) case-patients who provided information were considered to be immunocompromised (i.e., had an HIV infection, an organ transplant, a history of invasive cancer, and/or used systemic corticosteroids in the year before diagnosis).

Those with *C. gattii* infection were more likely than the general population to be >50 years of age (p<0.001), be current smokers (p<0.001), be infected with HIV (p<0.001), or have a history of invasive cancer (p<0.001) (Table 2). *C. gattii*–infected persons were more likely than the general population to belong to age groups >50 years (i.e., 50–59 years, 60–69 years 70–79 years, and >80 years). The incidence of *C. gattii* infection was highest in those 70–79 years of age (2.5/100,000 population). Male sex was not associated with infection (p = 0.198) (Table 2). Those with *C. gattii* infection were not more likely to have a history of COPD or asthma than were the general population. Although 30 (27.0%) of 111 patients with available information had used systemic steroids in the 12 months before diagnosis, no valid population controls were found for comparison.

**Table 2:** Comparison of risk factor prevalence among *Cryptococcus gattii*–infected patients and the general population, British Columbia, Canada, 1999–2007

Risk factor	Prevalence, % (95% confidence interval)		p value
	*C. gattii*–infected case-patients	General population	
Age >50 y	72.4 (64.9–79.8)	31.3	<0.001
Male sex	55.8 (46.4–65.1)	49.6	0.198
Current smoker	41.9 (35.3–48.6)	17.8 (16.9–18.6)*	<0.001
All invasive cancers	24.7 (21.7–27.7)	3.6	<0.001
Leukemia/lymphoma only	8.4 (7.6–9.2)	0.3	<0.001
Lung only	3.9 (3.3–4.5)	0.1	<0.001
HIV infection	3.7 (2.9–4.4)	0.2	<0.001
Chronic obstructive pulmonary disease	4.1 (−0.5 to 9.6)	8.0†	0.090
Asthma	3.4 (−1.1 to 7.9)	7.8	0.054

*From original data source. †Estimated based on range provided.

## Discussion

This controlled study identifies demographic, medical, and behavioral risk factors for *C. gattii* acquisition. Notably, ≈40% of *C. gattii*–infected patients were immunocompromised. This represents a departure from the prevalent view that *C. gattii* infects otherwise healthy persons. However, the proportion of immunocompromised patients remains lower than estimates of immunocompromise for patients infected by *C. neoformans*, many of whom have severe immunologic disorders (*26*).

In contrast to previously reported findings of an association between cryptococcal species infection and male sex, even when adjusting for HIV status (*14*), *C. gattii* patients in British Columbia were not statistically more likely to be male. It has been hypothesized that sex differences may be the result of differential environmental exposure during occupational or leisure activities. In British Columbia, sex differences are unlikely, given the widespread detection of the fungus in the environment, including urban and semiurban settings. Previous studies that have demonstrated sex differences were not species specific and likely included a disproportionate number of persons with *C. neoformans* infection; thus, gender may not influence acquisition of *C. gattii* specifically.

*C. gattii* patients in British Columbia were more likely than the provincial population to be >50 years of age. The relative absence of pediatric case-patients (4 patients in 9 years) confirms reports in other jurisdictions (*16*). This finding may have resulted from differential exposure, as suggested by serosurveys in Australia (*16*). In a New York study, however, most children had acquired antibodies to *C. neoformans* by the age of 5 years (*27*). Alternately, advancing age may be a risk factor simply because older persons are more likely to have medical risk factors for *C. gattii* infection or have had their immune function decline with age. In the case–control analysis, no significant difference in age was detected between case-patients and controls; therefore, this relationship could not be studied. The lack of age association between case-patients and controls may have been because of bias toward the inclusion of older controls because they were selected on the basis of a recent chest radiograph showing no abnormalities—a procedure more likely to be routinely performed in older patients.

In the case–control study, *C. gattii–*infected case-patients were significantly more likely than controls to have received systemic corticosteroid therapy. Increased susceptibility to cryptococcal infection after corticosteroid therapy has previously been described (*28*). Mouse experiments by Levine et al. in the 1950s demonstrated that mice, which usually were able to localize an induced subcutaneous cryptococcocal infection, could not do so after an intraperitoneal injection of 2.5 mg of cortisone acetate (*10*). Among US patients with culture-confirmed cryptococcal meningitis, persons who had received >20 mg of prednisone or who had Hodgkin lymphoma or other lymphoreticular malignancies were at significantly higher risk of dying than those without these conditions (*11*). In another case series of *Cryptococcus-*infected patients, disseminated disease developed in 59% of those treated with corticosteroids (*12*). More recently, corticosteroid use has been identified as a risk factor for disseminated disease in HIV-negative patients with pulmonary cryptococosis (*29*). Because oral steroids are sometimes used to treat cryptococcal infections, these findings present a medical challenge.

In the case–control study, a history of pneumonia or other lung conditions (including emphysema, chronic bronchitis, COPD, sarcoidosis) was associated with cryptococcosis. Pneumonia alone may not be an independently associated risk factor because it is associated with many lung conditions. Certain respiratory conditions, including bronchial obstruction, predispose the patient to recurrent bacterial pneumonia (*30**,**31*), and bronchopulmonary disorders may predispose a person to pulmonary cryptococcosis (*32*) because airway damage leads to a decreased barrier to infection. *C. gattii–*infected case-patients were not more likely than population controls to have experienced COPD, when that factor was examined alone. Notably, asthma was not identified as a risk factor by either the case–control or population-level analyses, although this condition, along with COPD, has been suggested as a risk factor (*31*).

Invasive cancers of all types were significantly more common in *C. gattii–*infected patients than in the general population. This finding held true for subgroups of patients with lung cancer and leukemia/lymphoma. Lymphoproliferative malignancies have traditionally been considered risk factors for cryptococcal infection and disseminated disease (*14*), and this appears to remain true for *C. gattii*–infected populations specifically. Although invasive cancers likely lead to an increased risk for infection, *C. gattii* infection may have been an incidental finding in patients with lung cancer, with the detection of the former facilitated through imaging and invasive procedures used for diagnosing the latter. In the case–control study, MORs also suggested that odds of infection were elevated for those with a history of invasive cancers; however, this difference was not significant.

During 1999–2007, six cases of culture-confirmed *C. gattii* infections were documented in HIV-positive residents of British Columbia. Although such cases are recognized (*33**,**34*), HIV infection has generally been associated with *C. neoformans* infection. This study highlights that HIV infection, although rare in *C. gattii* patients, is significantly more likely to be found in this patient group than in the general population. In fact, the strength of this relationship may have been underestimated: since HIV-positive persons with cryptococcal infection of undetermined species were excluded from the case definition, some *C. gattii* infections among HIV-positive patients may have been missed. Although the specific reason is unknown, HIV status may be associated with *C. gattii* infection because of differences in strain pathogenicity and host immune response.

Three outdoor activities—chopping wood, pruning, and cleaning up branches—were protective against infection. The reason for this is not clear because these activities would potentially have exposed the person to *C. gattii* in trees. Ongoing low-level exposure possibly results in immunity, preventing symptomatic infection if the person is exposed to a higher dose. More likely, these more vigorous activities are undertaken by healthy persons and reflect a general state of wellness rather than a protective effect of these specific behaviors. In contrast, outdoor building repairs represented a risk factor for infection. This may have been due to the disturbance of rotting wood colonized with *C. gattii*. Environmental experiments in British Columbia have demonstrated substantially increased airborne concentrations of *C. gattii* when colonized trees are felled or chipped (*35*).

Population comparisons suggest that significantly more *C. gattii–*infected case-patients are current smokers. Because smoking compromises the protective barriers in airways, it has been linked to increased risk for many lung infections (*36*). A previous study of AIDS patients with *C. neoformans* infection, which identified smoking as a risk factor for disseminated disease, suggested that fungus may be inhaled along with smoke particulates and deposited in small airways (*17*). Air-sampling studies of *C. gattii* in British Columbia have found that the airborne propagule is sufficiently small to be deposited in the upper lung (i.e., from 3.3 μm to >7 μm (*37*).

This study has several limitations. First, to ensure that asymptomatic *C. gattii*–infected patients were not included as controls, we required controls to have had chest radiographs showing no abnormalities. A selection bias may have been introduced, which could have reduced the likelihood of showing significant differences among variables, such as smoking, that might be associated with having a radiograph taken, leading to more conservative estimates or an inability to detect true differences in exposures between case-patients and controls. Similarly, the small sample size of the case–control study may have limited its power to detect significant exposures. Therefore, when possible, population comparisons were also performed to validate case–control findings.

In addition, risk factors for both analyses were collected by self-report, which may have been inaccurate. Also, the case–control analysis examined risk factor information in the 3 months before disease onset. Subsequent research has shown that the incubation period for this disease is typically longer (*38*). This long incubation period may have affected the study’s ability to accurately detect behavioral and environmental risk factors; medical risk factors were unaffected because these were reported as “ever diagnosed with.” Despite a case definition specifically designed to exclude case-patients infected with *C. neoformans*, some misclassification may have occurred, given that only 50% of infections in case-patients included in the case–control study and 57% of infections in case-patients included in population comparisons could be confirmed by laboratory typing.

## Conclusion

Although most cases of *C. gattii* infection do occur in otherwise healthy persons, our findings suggest that infection is associated with immunosuppressive states induced by oral corticosteroid use and invasive cancers as well as with weakened pulmonary function resulting from previous lung infections and smoking (Table 3). Despite HIV infection being rare among *C. gattii–*infected case-patients, it also occurred more frequently in infected persons than in the general population. Recent evidence indicates that *C. gattii* is now spreading in the Pacific Northwest of the United States (*19**,**39*). Physician awareness of risk factors should assist with diagnosis of this serious but treatable infection in areas where the disease is emerging.

**Table 3:** Risk factors for *Cryptococcus gattii* infection as evaluated by case−control study and population-based comparison, British Columbia, 1999–2007*

Risk factor	Case−control study†			Population comparison‡	
	Evaluated	Association with disease		Evaluated	Association with disease
Age	Yes	None		Yes	Positive
Sex	No			Yes	None
Oral steroid use	Yes	Positive		No	
Invasive cancer	Yes	None		Yes	Positive
Pneumonia	Yes	Positive		No	
Other lung problems§	Yes	Positive		No	
COPD	No			Yes	None
Asthma	Yes	None		Yes	None
Current smoker	Yes	None		Yes	Positive
HIV infection	No			Yes	Positive
Environmental exposures	Yes	Negative¶		No	

*COPD, chronic obstructive pulmonary disease. †n = 30 matched pairs. ‡n = 218 case-patients with *C. gattii* infection. §Includes emphysema, chronic bronchitis, COPD, sarcoidosis. ¶Negative associations were found for cutting/chopping wood, pruning, and cleaning up branches.
